# Challenges and recommendations for high quality research using electronic health records

**DOI:** 10.3389/fdgth.2022.940330

**Published:** 2022-08-19

**Authors:** K Honeyford, P Expert, E.E Mendelsohn, B Post, A.A Faisal, B Glampson, E.K Mayer, C.E Costelloe

**Affiliations:** ^1^ Global Digital Health Unit, School of Public Health, Imperial College London, London, United Kingdom; ^2^ Health Informatics Team, Division of Clinical studies, Institute of Cancer Research, London, United Kingdom; ^3^ Global Business School for Health, University College London, London, United Kingdom; ^4^ Department of Computing, Imperial College London, London, United Kingdom; ^5^ UKRI Centre for Doctoral Training in AI for Healthcare, Imperial College London, London, United Kingdom; ^6^ Chair in Digital Health, Faculty of Life Sciences, University of Bayreuth, Bayreuth, Germany; ^7^ Department of Bioengineering, Imperial College London, London, United Kingdom; ^8^ Translational Data Analytics and Informatics in Healthcare, Department of Surgery & Cancer, Imperial College London, London, United Kingdom; ^9^ Imperial Clinical Analytics, Informatics and Evaluation (iCARE), NIHR Imperial BRC, Imperial College Healthcare NHS Trust, London, United Kingdom; ^10^ Health Informatics Team, Royal Marsden Hospital, London, United Kingdom

**Keywords:** research ethics, data quality, electronic health records, trusted research environment, digital health, research protocol, real world data

## Abstract

Harnessing Real World Data is vital to improve health care in the 21st Century. Data from Electronic Health Records (EHRs) are a rich source of patient centred data, including information on the patient's clinical condition, laboratory results, diagnoses and treatments. They thus reflect the true state of health systems. However, access and utilisation of EHR data for research presents specific challenges. We assert that using data from EHRs effectively is dependent on synergy between researchers, clinicians and health informaticians, and only this will allow state of the art methods to be used to answer urgent and vital questions for patient care. We propose that there needs to be a paradigm shift in the way this research is conducted - appreciating that the research process is iterative rather than linear. We also make specific recommendations for organisations, based on our experience of developing and using EHR data in trusted research environments.

## Introduction

A vast quantity of Real Word Data (RWD) are sitting in health providers servers, and harnessing these is recognised as vital to improving health systems and services, but access and usage is still difficult. We need to improve data access and centralisation. The United Kingdom (UK) has the opportunity to demonstrate the power of EHR research on a large scale. Universal, taxpayer funded healthcare is accessible to everyone living in the UK, which is centrally planned and delivered as the National Health Service (NHS). Importantly, within the NHS is “NHS digital”, which sets a national strategy for technologies and data within healthcare. In theory, this could allow for a national, coherent and integrated data strategies, a centralised data repository and universal streamlined access for research. However, to maximise patient benefit from RWD, we need to create a cross-sector environment that fosters synergy between researchers, clinicians and health informaticians, to ensure state of the art methods can be applied to answer relevant questions and have impact in clinical practice (1).

The use of routinely collected healthcare data in research has proliferated over the last 10 years; a search for “real world data” on PubMed shows an increase in publications from 353 in 2009 to 8,370 in 2021. In the UK, EHRs are pivotal to NHS Digital's strategy; who envisage routinely collected data being used to maximise accessibility and quality of healthcare, the development of research and new digital products (2).

During the COVID-19 global pandemic the urgent need to use RWD data to inform decision making became all the more evident (3). In addition to its use in direct patient care and capacity planning, RWD are needed in order to understand the complex relationships surrounding external shocks to health systems, such as the current pandemic (4).

We use Electronic Health Records as an umbrella term for any information pertaining to patient care which is recorded in digital format. They are collected from sources including electronic patient records (EPRs), financial records and disease registries and might or not be joined together to produce a unified view of patients health (5). Increasing the integration of EHR across systems and platforms provide a comprehensive view of patients across multiple health providers, maximising the benefit to patients.

Researchers have extensive experience of producing high quality research from patient data, and we have worked with approval bodies which have adapted protocol guidelines to support this work. However, EHRs are different to many other sources of patient data; they are neither an opportunistic collection of existing administrative data sources nor a purposefully designed comprehensive single database (registry) (6). Rather, they collate information on the patient's clinical condition, laboratory results, diagnoses and treatments as they are experiencing health care. They thus reflect the true state of a health system, making them an important asset to research, service evaluation and quality improvement, provided an adequate analysis framework is in place.

Research using EHRs can draw on a wide variety of data, and the high frequency of observations captured makes EHRs a candidate for Big Data Solutions (7). For example, EHR data have been used to reduce risk of mortality through alerts (8), predict hypoglycemia (9), show that increased intra-hospital movement is associated with odds of hospital acquired infection (10), and enable contact tracing of patients within hospitals (11). EHR data also have the potential to support clinical decision making through the development of artificial intelligence (AI) algorithms (12). Finally, EHRs can also be used to understand large scale impacts of interventions and external influences on the health system in real-time, such as changes in emergency attendance in England in response to the COVID pandemic and vaccine uptake (13–15).

Much has been written about various aspects of harnessing EHR data for research, including the RECORD statement, which provides clear guidance on best practice for reporting studies using routinely collected observational data, (16). Nonetheless there is limited guidance on how this best practice can be achieved and few authors have considered these issues together, and highlighted their interdependencies.

Electronic Health Records have significant challenges associated with their use, including: the potential for poor data quality (17) complicated privacy and ethico-legal considerations (18, 19) ensuring bias in data is well understood (6); use of appropriate statistical methods to take into account missing or irregular data points (20). These issues must be considered together and their interdependencies highlighted, understood and taken into account when designing and ethically assessing research protocols, platform for access and knowledge that the results will be generalisable and it will be possible to validate the data.

In this paper, we draw from our extensive personal experiences of using Trusted Research Environments (TREs) containing data from EHRs and the challenges that we encountered. We provide a summary our learnings associated with accessing EHR data for large-scale data projects and make recommendations for developing a framework to enable access to data to facilitate high-quality patient-centric research.

## Challenges associated with creating an ecosystem for high quality research using EHRS

Accessing, operationalising and utilising EHR data for public health and health systems research present specific challenges. Here, we highlight five key themes we believe are vital to producing high quality research using data from EHRs.

• Developing the research protocol
• Access and ethical approval
• Data quality
• Analysis platform
• Generalisability and research integrity

### Developing the research protocol

The development of the research protocol is crucial to gain funding, ethical approval and achieve stakeholder engagement. EHR data are not prospectively collected, and the researcher does not collect specific clinical information. Even mature EHR data sets are unlikely to have highly descriptive metadata for each data element, so the development of prospective research protocols that determine which data will be collected, eligibility criteria, endpoints or outcomes and power calculations, is not feasible. In order to answer specific hypotheses and research questions, significant focus on exploratory and descriptive analysis is required before data selection can be finalised. Data exploration, in conjunction with clinicians and health informaticians, needs to be conducted to understand the data quality and agree on variable definitions. This is the case for observational association and retrospective cohort studies, but also for answering causal questions (21).

### Recommendation - developing the research protocol

**Figure 1:**
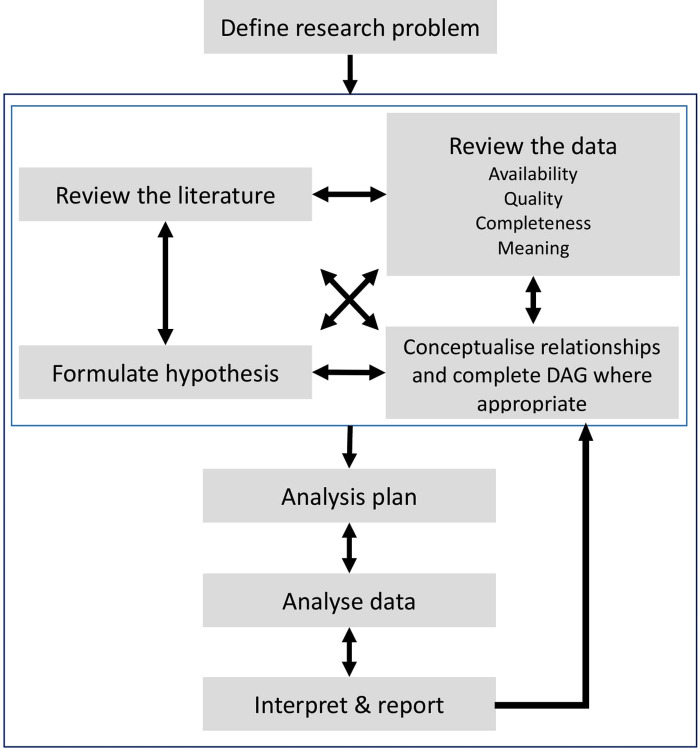
Schematic representation of the adaptive process when conducting research using retrospective data from Electronic Health Records. An iterative process is recommended between the preliminary stages of a study highlighted by the light grey square (reviewing the literature, formulating hypotheses, data exploration and conceptualising its relationships) and the later stages of developing analysis protocols, interpretations of results and final reports.

We believe that the use of EHR data for research needs a paradigm shift in the way research is conducted; moving away from a linear, prespecified process to an iterative approach which is developed within multidisciplinary teams of researchers, clinicians and health informaticians, see Figure 1. This type of approach implies that, whilst we support the pre-registration of observational studies (22), an adaptive approach to data selection and proposed analysis is key when developing research protocols using EHR data, which allows research protocols to become more specific over time as more is understood about the data, its quality and the best analysis pipeline.

### Access and ethical approval

In the UK, the majority of European countries, the US (23), China (24) and Japan (25) patient consent for research using de-identified routinely collected data, such as those within EHRs, is not required. In Europe the General Data Protection Regulation 2016/679 (GDPR) explicitly includes the processing of personal health data “for reasons of public interest”(26) and many accept the use of data collected for patient care should be used for health research and health system quality improvement (27). For this paradigm to continue, it is imperative that high quality research is demonstrably conducted for patient benefit and in the public interest whilst maintaining patient privacy and confidentiality. However, Goldacre et al (28) have highlighted that current data access and ethical approval processes can lead to projects being abandoned. Large investments in data collection are fruitless if the bar to access the data is too high.

We argue that when using data from EHRs the research team and the governance body will need to consider whether the scope and quality of the data is likely to enable the question to be answered. It is important that governance bodies have a clear understanding that data requests may need to be refined as a result of the exploratory analysis, highlighted in the previous section, and that this provision is built into the assessment process, lest quality research opportunities and time are squandered. Templates and forms will need to reflect the nature of research using EHRs; traditional requirements, such as patient recruitment targets, adverse event details, and sample size and power calculations may not be appropriate, at least before the initial data assessment has been conducted (29). In addition, there should be greater emphasis on whether the application has included a description of data quality, and the steps that will be taken to determine data quality and that all possible fields pertaining to clinical values of interest have been identified.

Potential biases need to be carefully considered when using EHRs for research, lest they exacerbate existing imbalances present in healthcare delivery. The embedding of biases in statistical learning based automated pipeline is not limited to healthcare and exist in any setting where the training data is not representative of the target population (30). This must be carefully considered when assessing data availability, generalisability and applicability of the findings (31). Many research protocols for access and ethics committees are not yet specifically addressing this area, despite significant attention in terms of implementation (32–34). Importantly, EHR data itself can be inherently biased, for example from the data collection process mandated by the software or if the primary use of the data is for administrative or billing purposes (6).

Finally, the research summary and/or protocol submitted to the data governance body/ethical committee must clearly demonstrate that confidentiality will be preserved, that the research question is important and that the research team have the necessary skills to answer this question.

### Recommendation - access and ethical approval

Sufficient expertise is needed within access committees in order to review the data quality and sufficiency requirements. Currently there is an emphasis on clinician sponsorship of projects, often with an emphasis on senior rather than practicing, we believe that projects need the involvement of practicing clinicians, who can verify the fields and modes of entry of clinical observations. For example, specific medical diagnoses can be encoded in numerous ways: through diagnostic codes, within free-text fields and inferred by particular medications. This quirk within medical data has been described as a computable phenotype by Goldstein et al. (20). In addition to medical expertise, statistical and methodological expertise are critical for successful research. This multidisciplinary must be reflected in the composition of the ethics review panel.

### Data quality

Critical to all aspects of the research is the quality of data. EHRs contains two types of data: fields whose values are entered in the system in predefined boxes, also known as structured data, and unstructured data, such as free text. Free text represents a colossal amount of information and the treatment to structure this data using Natural Language Processing tools come with its own challenges (35–36). For the purposes of this paper, we focus on the quality of structured data, irrespective of its original source. There is an increasing body of knowledge of general principles for data quality within accepted domains (16, 37). The data entered into patients' EHRs needs to be credible, complete, available for all patients, current and using a uniformised reference language (38). Data is quality checked at various points in the hospital data reporting process, particularly if it is associated with reimbursement and external reporting, and often uses national and internationally recognised codes. However, data quality issues may persist as these checks are not necessarily focused on the research integrity of the and the majority of published studies relying upon EHR data do not report data quality limitations (39).

Many factors contribute to quality issues in real world patient data, which are well documented; for example errors can occur when clinical observations are entered by busy frontline staff (40–41). Furthermore, the potential for data “missing not at random” requires consideration in EHR data, as imputation methods may lead to biased results (42). The handling of EHR observations therefore needs careful consideration, as simple heuristic checks can lead to downstream biases (43).

All data issues can be compounded when healthcare providers use different EHR systems. Non-clinical researchers must work closely with health informaticians to adapt the complex logic needed to amalgamate multiple data sources captured in different clinical IT systems, in order to ultimately create a system-agnostic EHR data set. Initiatives, such as the OMOP Common Data Model introduced by the Observational Health Data Science and Informatics, aim at providing a unified representation and data format from disparate sources (44).

Finally, when EHR data is available to researchers its associated data dictionary typically includes field type, definition source and linkage information. However, in our experience, data dictionaries do not typically contain information on the quality of the data itself, plausible ranges and clinical meaning. This further emphasises the need for iterative research protocol development'.

### Recommendation – data quality

It is therefore essential that the expertise of clinicians, non-clinical researchers and health informaticians is collaborative so that a virtuous cycle of improvement in the quality, credibility and presentation of the data exist to ensure data quality and understanding increases, and facilitate future research projects.

In addition, we would recommend that ongoing research projects contribute to improving data dictionaries, and code resources for data cleaning.

One of the end goals is data integration across platforms, trust and countries, an international standard for data representation, such as the one developed by Observational Health Data Science and Informatics needs to be developed and widely adopted (45).

### Analysis platforms

EHR data is increasingly being accessed through cloud-based TREs, where researchers analyse data directly within secure systems, obviating the need for data export (28, 46). Integrated analysis platforms exist within TREs, and must consider both user experience and planned research; different skill sets has been identified as a key factor affecting data use (47). The analysis environment should therefore be easy to use, accommodating varying levels of computing ability, or provide access to professional services to carry out the analysis. The hardware and software available must be versatile to facilitate projects including small scale service evaluations, which may need a “point-and-click” self-service tool, e.g. software suits like Excel, SPSS or Tableau, for researchers that want to carry out small research projects but do not have the necessary programming skills or professional service analysts that can mediate access (47). Provisions also need to be made to support more sophisticated analysis and big data projects, undertaken by “power-users”, including programming languages such as Python or R and direct data access with SQL (47).

A key challenge with EHR data is that it is not organised for research purposes and needs considerable processing (20), our experience suggests that for “power-users”; highly modular structure of simple linkable tables *via* de-identified patient and event identifiers is advantageous. This allows for tailored access to data based on project needs, accelerates database queries and minimises database load. Access to the database interfacing directly with the analysis environment is hugely beneficial; as it allows a direct exploration of the data. However, as many users might be unable to use query-based languages to prepare data for analysis, data extraction and preparation support should be provided and different costing models for this have been identified in the US (47).

### Recommendations – analysis platforms

Appropriate hardware infrastructure, including graphics processing units (GPUs) and parallel computing, should be considered to complement computationally intensive methods and the size of the data offered.

Training in using the platforms, analysis packages and software, which may have a very different “feel” to desktop computing, must be factored into the project lifetime, which is crucial given the time-limited nature of research funding.

To summarise, the analysis environment must be professionally and actively maintained for performance for a range of users, be flexible to allow for easy installation of new and updated software packages, and cater for the evolving needs of ongoing projects. Software version control systems should be available to allow trackability and reproducibility of research projects.

### Generalisability and research integrity

Data from single healthcare settings, such as one hospital or a single GP surgery, means that results are interpreted clearly within a local context, however, the generalisability of the results may be limited. Many factors will affect generalisability, Ghassemi et al (48) highlight local hospital practices, different patient populations, available equipment and the specific EHR in use. Important insights will therefore be generated by understanding commonalities and differences across healthcare settings (49). In the UK, government funded initiatives, such as the National Institute of Health Research Health Informatics Collaborative (NIHR-HIC) have facilitated combination of EHR data across NHS hospitals, allowing for sampling of larger, more generalisable populations (50).

In addition, machine learning and the development of predictive or rapid risk stratification algorithms is becoming increasingly common within EHR data. Validation is a key requirement for these algorithms and may require research to be applied to similar datasets in other healthcare settings. However, while the analysis code itself should be portable, the preparation of the data to achieve the correct input format is likely to be system specific and challenging to share with other researchers. Sharing machine learning code may also lead to issues with data security which have not yet been widely discussed. For example, some ML algorithms, such as support vector machines, contain samples of the data itself and may allow re-identification, which needs to be understood before code is shared openly (51).

EHR data analysis must also be reproducible and transparent to maintain research integrity. While open data is not a viable model for healthcare data, tools must be put in place to ensure results and data can be checked independently and data access made available to external researchers. We advocate working towards a model which allows automated methods, including federated machine learning, where the data stays local. This will necessitate common standards for data interoperability (52–53). Finally, it is essential that the move to vendor-provided EHR systems does not impede researchers' access to data and or the dissemination of research findings through publication (54).

## Discussion

EHRs are a valuable resource for research, but the *current* frameworks may not be well suited to handle the associated challenges we have detailed above. There is clear association and intersectionality between the challenges and recommendations; no individual recommendation stands alone, and they are all interdependent, e.g. in order to derive a study protocol, the data quality needs to be understood. A paradigm shift is needed in how we plan, approve and value EHR data-based research. Importantly, every research project must firstly ask the following questions:

> Is the research question clinically important and likely to lead to improved patient and/or public health?
> Can the data available answer the question?
> Is the proposed methodology appropriate, given the research questions and the data available?

These questions can only be answered by a multidisciplinary team. As such, patients, clinicians, statisticians, and health informaticians are all **equally** vital in planning, approving and performing high quality research. Our recommendations are summarised below, and are key to making progress in effective and high quality research using EHRs.

### Adapt research design and associated approval processes to work effectively with EHR data

• While transparency and clear plans prior to data examination is paramount (55), a flexible approach to data selection and analysis is needed for EHR research. The population sample, data fields extracted and planned statistical analysis may need to be modified as understanding of data quality is developed. We believe that an iterative approach to analysis can be a valid scientific process if all decisions and rationale are documented in detail. This paradigm is already widely accepted in qualitative research traditions, where data is revisited as understanding of the dataset deepens, novel connections are made and additional questions emerge (56–57). The data should therefore be considered as a dynamic set of information.
• Data access committees and ethics boards need to adapt their processes to research of this kind, by focussing more on data security and results validation, with less emphasis on participant eligibility and adverse event monitoring. This should be added to the recommendations given by Goldacre et al. (28).
• As data quality and composition is explored, and understanding of the data increases, necessary changes should be documented and implemented, ultimately increasing data value and usability.

### Ensure a strong research team with the right mix of skills and collaboration

• High-quality research needs effective collaboration between clinicians, non-clinical researchers and health informaticians. The partners need to work synergistically, have open channels of communication and ensure all members have capacity to actively engage in the project when their expertise is needed.
• In order for health informaticians, clinicians and non-clinical researchers to work collaboratively on a potentially dynamic dataset there needs to be an integrated access and analysis platform.

### Uphold research integrity

Reproducibility and open science are vital for research integrity and validity. Mechanisms allowing independent scrutiny of data, analyses and in-house developed software should be built into platforms enabling research using EHRs, without compromising data management, confidentiality and intellectual property.

## Conclusion

To conclude, we want to remind our readers that TREs, data access, AI are tools not goals in the realm of healthcare (58). The success of the “big data approach” in healthcare will not be measured by number of secure environments, size and number of data sources or the amount of greenhouse gas emitted, but by the significance of the improvements of patients outcomes. While there is no such thing as a free lunch, we believe that an equalitarian distribution of power and influence among clinicians, informaticians and statisticians of all disciplines is the shortest path to success.

This approach will support major improvements to health care, allow more rapid responses to health care crises and foster improved collaborations between health informaticians, clinicians and non-clinical researchers. We have a responsibility to ensure that data is used to improve patients' health outcomes.

## Data Availability

The original contributions presented in the study are included in the article/Suplementary Material, further inquiries can be directed to the corresponding author/s.
